# The Effect of High Selenite and Selenate Concentrations on Ferric Oxyhydroxides Transformation under Alkaline Conditions

**DOI:** 10.3390/ijms22189955

**Published:** 2021-09-15

**Authors:** Michaela Matulová, Marek Bujdoš, Marcel B. Miglierini, Martin Cesnek, Eva Duborská, Katarína Mosnáčková, Hana Vojtková, Tomáš Kmječ, Július Dekan, Peter Matúš, Martin Urík

**Affiliations:** 1 Institute of Laboratory Research on Geomaterials, Faculty of Natural Sciences, Comenius University in Bratislava, Mlynská Dolina, Ilkovičova 6, 84215 Bratislava, Slovakia; michaela.matulova@uniba.sk (M.M.); marek.bujdos@uniba.sk (M.B.); eva.duborska@uniba.sk (E.D.); peter.matus@uniba.sk (P.M.); 2 Institute of Nuclear and Physical Engineering, Slovak Technical University in Bratislava, Ilkovičova 3, 81219 Bratislava, Slovakia; marcel.miglierini@stuba.sk (M.B.M.); julius.dekan@stuba.sk (J.D.); 3 Department of Nuclear Reactors, Faculty of Nuclear Sciences and Physical Engineering, Czech Technical University in Prague, V Holešovičkách 2, 18000 Prague, Czech Republic; martin.cesnek@fjfi.cvut.cz; 4 Polymer Institute, Slovak Academy of Sciences, Dúbravská Cesta 9, 84541 Bratislava, Slovakia; katarina.mosnackova@savba.sk; 5 Department of Environmental Engineering, Faculty of Mining and Geology, VŠB—Technical University of Ostrava, 17 Listopadu 15/2172, 70800 Ostrava-Poruba, Czech Republic; hana.vojtkova@vsb.cz; 6 Faculty of Mathematics and Physics, Charles University, V Holešovičkách 2, 18000 Prague, Czech Republic; kmjec@mbox.troja.mff.cuni.cz

**Keywords:** ferric oxyhydroxides, Mössbauer spectroscopy, selenium, sorption

## Abstract

Iron-based nanomaterials have high technological impacts on various pro-environmental applications, including wastewater treatment using the co-precipitation method. The purpose of this research was to identify the changes of iron nanomaterial’s structure caused by the presence of selenium, a typical water contaminant, which might affect the removal when the iron co-precipitation method is used. Therefore, we have investigated the maturation of co-precipitated nanosized ferric oxyhydroxides under alkaline conditions and their thermal transformation into hematite in the presence of selenite and selenate with high concentrations. Since the association of selenium with precipitates surfaces has been proven to be weak, the mineralogy of the system was affected insignificantly, and the goethite was identified as an only ferric phase in all treatments. However, the morphology and the crystallinity of ferric oxyhydroxides was slightly altered. Selenium affected the structural order of precipitates, especially at the initial phase of co-precipitation. Still, the crystal integrity and homogeneity increased with time almost constantly, regardless of the treatment. The thermal transformation into well crystalized hematite was more pronounced in the presence of selenite, while selenate-treated and selenium-free samples indicated the presence of highly disordered fraction. This highlights that the aftermath of selenium release does not result in destabilization of ferric phases; however, since weak interactions of selenium are dominant at alkaline conditions with goethite’s surfaces, it still poses a high risk for the environment. The findings of this study should be applicable in waters affected by mining and metallurgical operations.

## 1. Introduction

Selenium naturally occurs in oxidation states of −II, 0, IV and VI, with selenites and selenates being prevalent in oxic environments [1,2,3]. Since it usually occurs in a wide range of pHs as oxyanion, selenium is considered a highly mobile element. Its environmental mobility and bioavailability are therefore one of the concerns of environmental toxicologists, since it is essential for organisms, yet is highly toxic under certain conditions [4,5,6,7,8]. The primary natural geogenic sources of selenium are phosphatic, organic-rich sedimentary rocks [9], carbonate rocks and soluble salts in marine sediments [10]. The main anthropogenic sources include excavated rocks from coal mining, tunnel construction and underground space development [11,12]. It also includes coal combustion, nonferrous metal smelting, and agricultural runoff [13,14].

Iron-based nanoparticles are now being used in diverse applications, such as environmental remediation [15,16,17], and can be used in many other industries such as pharmaceutical sciences, biosciences and biotechnology [18,19]. Iron oxyhydroxides’ nanoparticles have a promising capacity for toxic ion uptake and hence are widely employed in the environmental remediation techniques. Based on the efficiency, applicability, and accessibility of iron compounds, the current research has studied iron co-precipitation for the purpose of removal of potential toxicants from industrial wastewaters. For example, co-precipitation with iron seems to be very effective for arsenic removal from wastewaters [20,21,22]. Furthermore, synthesized layered double hydroxides nanoparticles are promising for wastewater treatment [23,24]. For instance, Fe-Ti layered double hydroxide was successfully used for selenite removal from marine sediments and soil [25].

There is a huge variety of methods applicable for selenium removal from contaminated waters. However, the most effective ones include the step of co-precipitation using iron-based precursors [26,27,28]. In case of abrupt increase in pH during co-precipitation, the synthetic products of oxygenated ferric phases consist primarily of nanosized particles [29]. Ferrihydrite adsorption or iron co-precipitation is the technology recommended by US EPA as the best demonstrated available technology for selenium treatment [30]. Iron oxyhydroxide is formed when a ferric salt is added to the wastewater, while ferric hydroxide and ferrihydrite precipitate simultaneously and adsorb selenium on iron surface.

Merrill et al. [31] conducted a pilot scale study to evaluate the technical and economic feasibility for the removal of selenium and arsenic from ash pond effluents, while it contained 40 to 60 µg L^−1^ of selenium, which was removed by co-precipitation with amorphous iron oxyhydroxide in a range from 56% to 80% for iron dosages of 14 mg L^−1^ and above. The optimum removal was observed at pH 6.2 and below. In another study with an initial selenium concentration of 5 mg L^−1^, ferric oxyhydroxide flocs formed by the precipitation of ferric chloride was found to remove more than 95% of selenate using ferric chloride dose of 1 g L^−1^. Selenite was removed faster and required lesser material doses. Furthermore, for a dose of 7 g L^−1^ of ferric chloride more than 99% of selenate was removed with ferrous-ferric co-precipitation method [32]. Ferric sulphate has similar principle of selenium removal such as ferric chloride, however less efficiency to ferric sulphate was suggested [33]. Furthermore, iron precipitates most effectively at higher pH and metal contaminants are more efficiently removed at alkaline pH, as well [34]. The presence of selenium oxyanions might interrupt the removal of heavy metals by its impact on the iron oxyhydroxides crystallization.

The abrupt media alkalization to co-precipitate ferric phases seems less suitable for the removal of selenites and selenates, because of their low affinity towards hydrated ferric iron precipitates’ surfaces at neutral or alkalic pH regions [35,36]. Still, as a secondary effect, the presence of high concentrations of selenium oxyanions may significantly hinder the kinetics of ferric oxyhydroxide precipitation and crystal growth. It may even stabilize the products similarly to a reported decrease in size distribution of ferric particles co-precipitated in the presence of high concentrations of phosphates [37]. This process, however, can be beneficial for other element removals due to the increase in the sorbent’s surface area.

This inspired us to evaluate the effects of strongly alkaline conditions on oxygenated ferric phase transformations during their co-precipitation with highly concentrated selenium oxyanions. The 1:1 molar ratio of iron(III) and selenium was used, since our previous research indicated that only negligible changes in goethite’s iron is induced by low selenate concentrations [36]. To achieve these objectives, a Mössbauer spectroscopy, ATR-FTIR, SEM, and high energy X-ray diffraction (HEXRD) were applied on samples collected during co-precipitation and transformation of ferric oxyhydroxides in the presence of high concentrations of selenite or selenate. The results of our study might improve understanding of geochemical factors controlling selenium mobility in industrial wastewaters, uranium mill tailings, and at near field of the nuclear waste packages.

## 2. Results and Discussion

### 2.1. Transformation of Iron Precipitates under Alkaline Conditions

**Figure 1:**
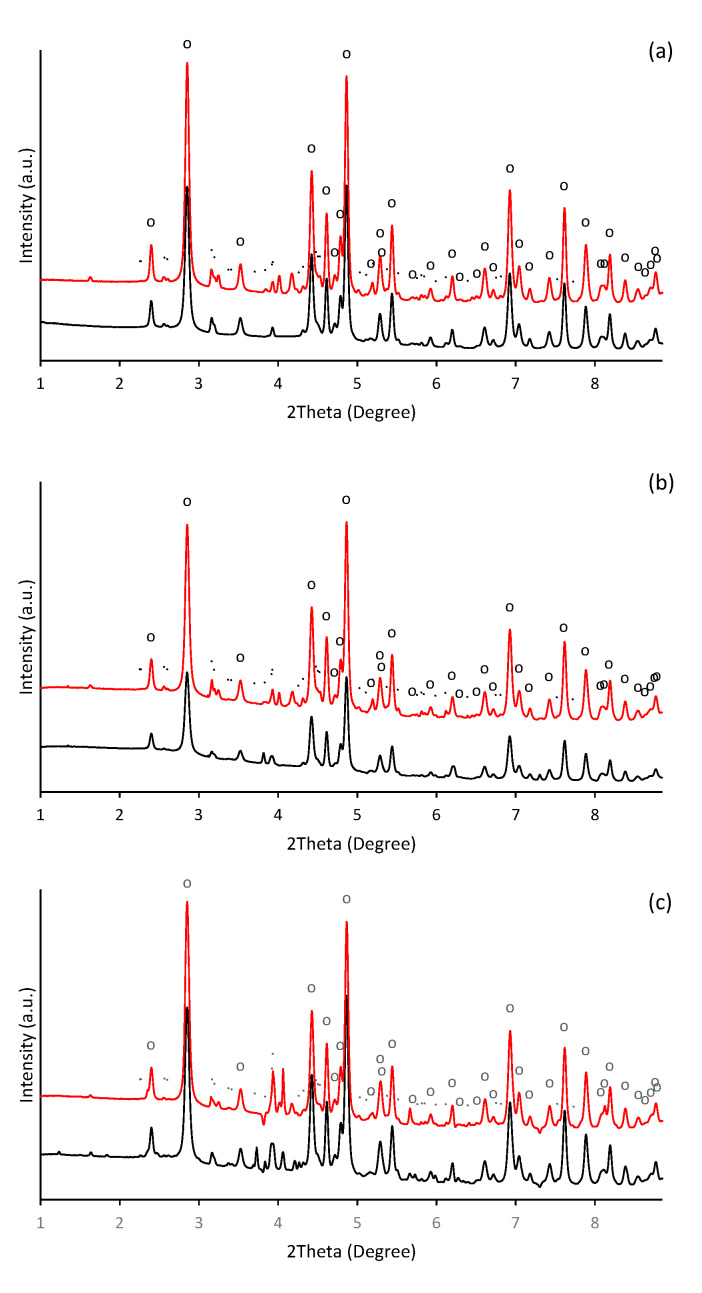
Diffraction patterns of the ferric precipitates crystallized in alkaline (**a**) selenium-free, (**b**) selenite-treated, and (**c**) selenate-treated solution on the 2nd (black line) and 5th (red line) day of precipitates maturation (“o” symbols indicate characteristic XRD patterns for goethite (α-FeOOH) and symbols of “.” indicate precipitated potassium nitrate (KNO_3_)).

In alkaline aqueous solutions, ferric iron readily hydrolyses and precipitates. Thus, the formation of aggregates was observable almost immediately after alkalization of ferric nitrate solution. During initial phases of precipitation in alkaline solutions, the poorly crystallized nanosized iron phase such as ferrihydrite (Fe_10_O_14_(OH)_2_) and ferric hydroxide (FeOH_3_) are usually formed [38,39,40], which subsequently transforms into more thermodynamically stable mineral phases as the maturation of precipitates progresses. Hence, in our experiment, the precipitate of thermodynamically stable goethite was identified one day after initiation of precipitation. It was confirmed by HEXRD analysis (Figure 1), which recognized the presence of goethite (α-FeOOH) as a major crystalline phase (refined lattice parameters: a = 4.618(3) Å, b = 9.973(5) Å, c = 3.027(2) Å) in a day-old precipitate (Figure 1a). It also indicated the presence of a crystal structure other than goethite.

Beside the ferric iron minerals, some authors have identified in ferric precipitates spectral patterns that can be attributed to the precipitated background electrolyte [41]. While it was less probable than the presence of the metastable 2-line ferrihydrite (the goethite precursor phase [42]), or 6-line ferrihydrite, which is a likely intermediate product during the conversion of 2-line ferrihydrite to goethite [43,44], the precipitates of potassium nitrate were identified (Figure 1a). In our experiment, most probably the ferrihydrite precipitates appeared in an earlier stage. Furthermore, not all peaks were assigned; thus, the occurrence of some unknown poorly ordered ferric precipitates are still possible.

**Figure 2:**
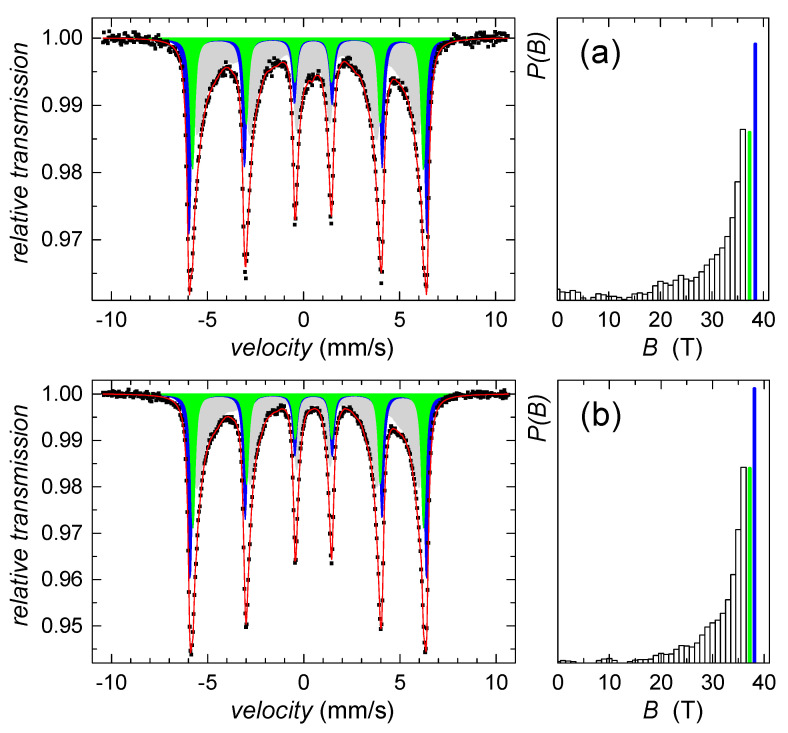
Mössbauer spectra (left) and corresponding *P(B)* distributions (right) of the control sample on (**a**) the 2nd day and (**b**) the 5th day of precipitates maturation. Distributed components are plotted in light grey color, crystalline subspectra are in blue and green color together with corresponding values of hyperfine magnetic fields.

Since we failed on identification of this sparse component using the HEXRD analysis, Mössbauer spectroscopy was utilized. According to the hyperfine Mössbauer parameters, the ferrihydrite was not identified, while goethite was confirmed. Mössbauer spectra in Figure 2 indicate the presence of well-established goethite structures as well as a goethite-like disordered phase. The former exhibits two sextets with hyperfine magnetic fields of 38 T and 37 T, isomer shifts of 0.37 mm/s, and quadrupole shifts of −0.27 mm/s, both. The disordered component was described by distribution of hyperfine magnetic fields, *P(B)*, featuring average values of 28.6 T and 31.2 T, for the samples collected on the second and fifth day of the precipitate maturation, respectively. Average isomer shift and quadrupole shift values were not changed with time and they were of 0.35 mm/s and −0.28 mm/s, respectively. However, relative content of the disordered component has decreased from 52% down to 48%, which suggests a gradual increase in the number of structurally better-established units with time of maturation.

All spectral parameters derived from Mössbauer analyses are listed in Table 1.

**Figure 3:**
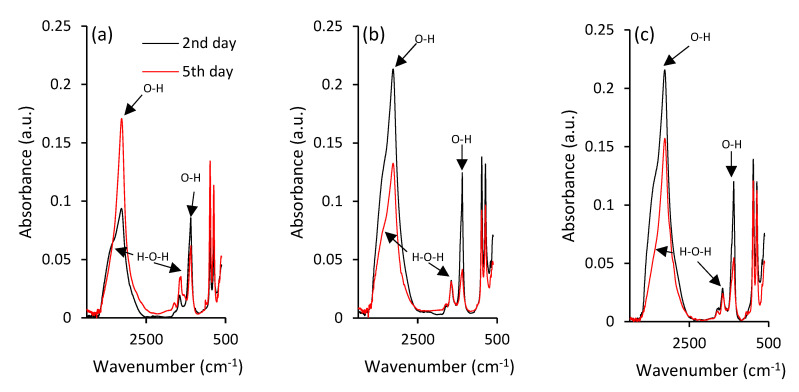
ATR-FTIR spectra of the ferric precipitates in alkaline (**a**) selenium-free, (**b**) selenite-treated and (**c**) selenate-treated solutions on the 2nd and 5th day of precipitates maturation.

Negligible structural changes and rearrangements of iron during maturation of ferric precipitates are also illustrated by ATR-FTIR spectra in Figure 3a. There are two strong absorbance bands at 885 and 796 cm^−1^ in the hydroxyl deformation region, labelled as δ(OH) and γ(OH), respectively, that can be assigned to goethite’s characteristic vibrations [45,46], and provide some information on its crystallinity and substitution in mineral structures [45].

Since the vibrational bands between 1800 and 1000 cm^−1^ generally belong to water bending modes, the bands observed at ~1650 cm^−1^ can be assigned to the bending vibration of weakly adsorbed molecular water [47]. However, it was suggested that the peak at ~1390 cm^−1^ corresponds to the structural hydroxyl groups at the surface of goethite [48]. The ATR-FTIR spectrum also shows the strong stretching vibration band at approximately 3129 cm^–1^ [49] that can be attributed to bulk hydroxyls. The observed variations during maturation in the shoulder at approximately 3420 cm^−1^ could be attributed to surface-adsorbed water, since it is characterized as stretching H-O-H [50,51].

**Figure 4:**
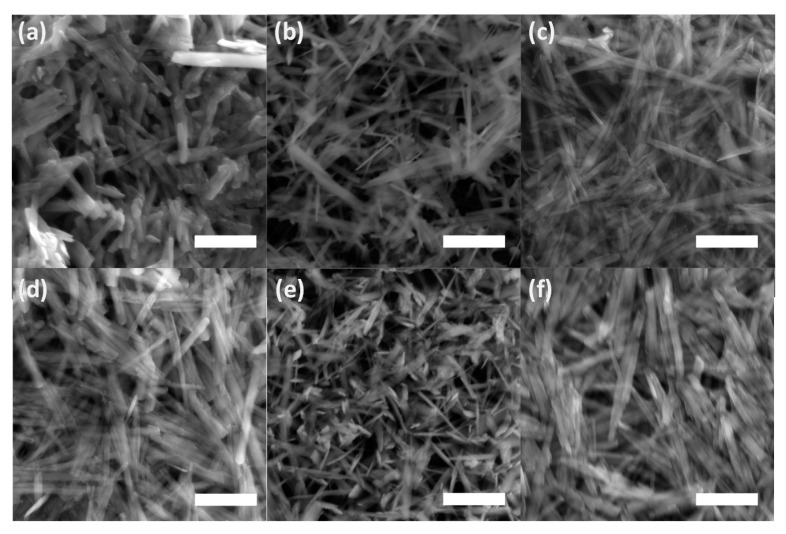
Scanning electron microscope (SEM) images of the ferric precipitates in alkaline (**a**) selenium-free, (**b**) selenite-treated, and (**c**) selenate-treated solution on the 2nd day of precipitates maturation; in (**d**) selenium-free, (**e**) selenite-treated, and (**f**) selenate-treated solution on the 5th day of precipitates maturation (white bars correspond to 1 µm).

The lines displayed at powder diffractograms in Figure 1 are relatively narrow, which might signal the formation of nanosized or minute crystals of both goethite and unidentified mineral components. This is supported by the direct examination of precipitates using SEM imaging in Figure 4. The individual flat elongated particles, which are observable in Figure 4a, are nanocrystals since their thickness is lesser than 0.1 μm. The final length and width in Figure 4d of individual crystals co-aggregated in bigger particles is ~2–3 µm and ~0.1–0.2 μm, respectively. These findings are in good agreement with observations of Das and Hendry [52].

### 2.2. Effect of Selenium Presence on Ferric Oxyhydroxides Transformation

Goethite growth is controlled by the chemical characteristics of ions present in its environment; thus, the adsorption and co-precipitation phenomena can inhibit and hinder the maturation process and kinetics of ferric precipitates’ transformation [53,54,55,56]. Selenium has also been shown to affect the formation of mineral phases since Börsig et al. [41] indicated that its interactions with ferrihydrite can alter the form of the final transformation product. However, our results agree with the previous observations only partially.

HEXRD analysis have confirmed the occurrence of goethite as a major crystalline phase in presence of selenite and selenate (Figure 1b,c); and their diffractograms exhibit almost identical patterns and characteristics as in the selenium-free treatment (Figure 1a). Still, the diffraction measurements of selenate-treated sample show noticeable presence of crystalline phase other than goethite. They are attributed to the precipitated KNO_3_. Thus, no significant deviations have been observed in the mineralogy by HEXRD analyses of ferric precipitates synthesized in the presence or absence of selenium. Although it becomes clear that the mineralogy of the system was not affected by the selenium, it seems to alter the morphology and the crystallinity of the goethite units during five day aging.

Figure 4e suggests a slight effect of selenite on goethite’s needle-like particles morphology during maturation in comparison to both selenium-free and selenate treatments (Figure 4d,f). The goethite grains in the presence of selenite appear to be slightly narrower and shorter, thus the increase in a unit volume during five day aging was lesser in comparison to selenium-free and selenate treatments. This observation has some practical implications regarding the mobility of co-existing ions. The changes in morphology during precipitates’ maturation can influence the adsorption of contaminants in natural environments since the surface area of the goethite varies due to different sizes and crystallinity of the goethite grains [52].

Further investigations using ATR-FTIR analyses highlighted the differences in goethite crystal grains between the selenium-free and selenium-treated samples. This was indicated by the absorbance peaks at 885 and 796 cm^−1^ in the hydroxyl deformation region of selenium-treated samples (Figure 3b,c), where the decrease of intensity did not follow the intensity increase in case of selenium-free treatment (Figure 3a). The opposite trends were observed for intensities of strong vibration stretching bands at 3129 cm^–1^ and stretching of the H-O-H shoulder at approximately 3420 cm^−1^ that are attributed to surface-adsorbed water [51]. Zhao et al. [57] hypothesized that the variations in amounts of chemisorbed water can contribute to observable differences in crystallinity of iron precipitates. Still, ATR-FTIR analysis did not provide any sound evidence for selenium-induced changes in ferric iron structure. Therefore, the Mössbauer spectroscopy was utilized.

**Figure 5:**
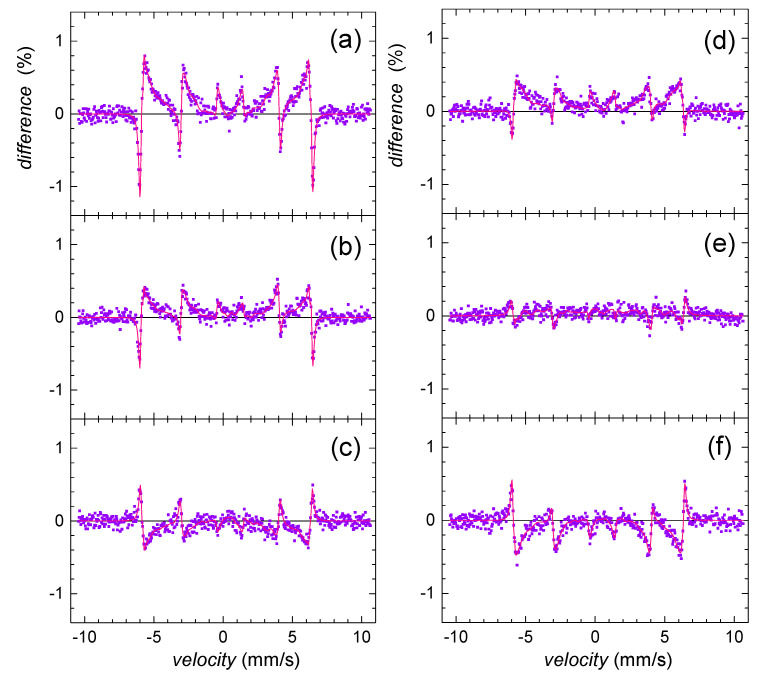
Differences between Mössbauer spectra of selenium-free control and selenite-treated sample (**a**,**d**); selenium-free control and selenate-treated sample (**b**,**e**); and selenite- and selenate-treated samples (**c**,**f**) as obtained on the 2nd (**a**–**c**) and the 5th (**d**–**f**) day of precipitate maturation.

Mössbauer analyses confirmed alteration of goethite in the presence of selenite and selenate in comparison with the selenium-free control. The most significant effect was observed at the initial phase, after 24-h. In order to visualize deviations in the corresponding Mössbauer spectra more clearly, the so-called difference spectra are presented in Figure 5. They were obtained by subtracting normalized spectra of the particular samples one from another at every velocity point. Note that the theoretically calculated curves are also provided. Deviations from the line of zero difference are most pronounced in Figure 5a. They highlight the impact of selenite in comparison with selenate (Figure 5b). Mutual comparison of these two is seen in Figure 5c.

The observed differences in Mössbauer data for selenium and selenium-free treatments imply the significant structural selenium-induced differences of iron in the goethite grains. Since Das et al. [58] reported that the arsenic oxyanions were capable of hindering the ferrihydrite transformation into goethite at pH 10, we assume that the selenium treatment did not cause permanent structural changes, but rather decreased the initial rate of goethite nucleation and growth. This phenomenon was more profound in the presence of selenite. Selenite probably more significantly retarded maturation of ferric precipitates and caused a less ordered system.

Wang et al. [59] reported that during aging of goethite in alkaline solutions, the distribution of the grain size became more uniform, and the small and flawed crystals with high potential energy disappeared because of the recrystallization. This implies that the precipitation system tends to increase the crystal integrity and homogeneity with time. This is in good agreement with our results since both selenium treatments seem to achieve a certain uniformity and stability that is characteristic for the selenium-free system. This is depicted in Figure 5d,e, where the observed differences in the Mössbauer spectra between the selenium-free control and both selenite and selenate treatments are less pronounced on the fifth day than at the beginning of the precipitation. Indeed, especially the effect of selenate dramatically decreases with time implying higher stability of the original selenium-free precursor against selenate treatment.

As demonstrated in Figure 5c,f, differences between the Mössbauer data of the selenite and selenate-treated samples did not significantly deviate with time. Consequently, we assume that the process of goethite maturation and growth is kinetically comparable for both treatments. Still, selenite affected the initial stage of goethite synthesis more significantly.

### 2.3. Effect of the Selenium on Thermal Transformation of Goethite to Hematite

Iron oxyhydroxides have a tendency to transform thermally due to their dehydration. In case of goethite, it can transform into the more thermodynamically stable hematite (α-Fe_2_O_3_). This transformation should affect the structural and morphological characteristics of ferric precipitates.

**Figure 6:**
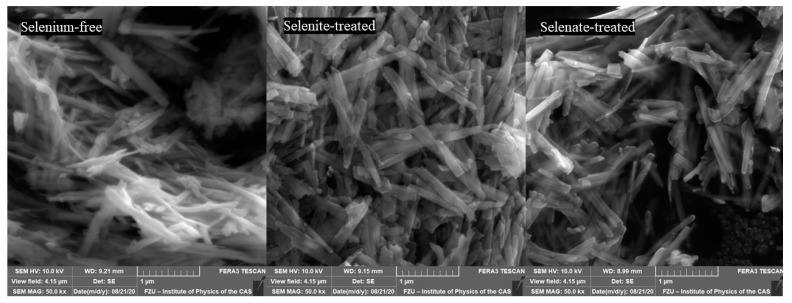
Scanning electron microscope (SEM) images of the ferric precipitates crystallized in alkaline selenium-free, selenite-treated, and selenate-treated solution after 5 days of precipitation and subsequent thermal transformation to hematite.

Gialanella et al. [60] reported that the shape and size of the newly formed hematite after treatment under 400 °C is almost the same as those of original goethite. Similarly, Figure 6 shows that hematite has basically the same habitus of elongated, prismatic grains as the goethite particles after dehydration at 250 °C. However, compared to goethite, hematite particles are fractured, most likely due to outgoing flux of the OH^−^ groups, which piled up internal stresses. 

**Figure 7:**
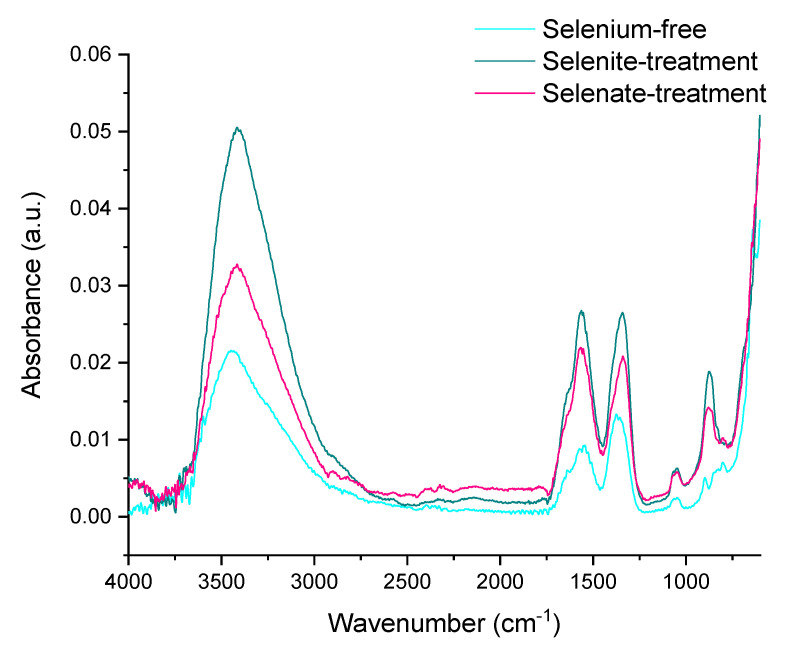
ATR-FTIR spectrum of the ferric precipitates crystallized in alkaline selenium-free, selenite-treated, and selenate-treated solutions after 5 days of precipitation and subsequent thermal transformation to hematite.

The loss of hydroxyl groups is clearly indicated by the ATR-FTIR analyses of synthesized hematite (Figure 7). The OH vibration stretching band at approximately 3129 cm^–1^ is not visible for any of the treatments. This signalizes the successful transformation of ferric hydroxide to oxide via loss of stoichiometric and non-stoichiometric hydroxyl units from goethite at low temperatures [61].

**Figure 8:**
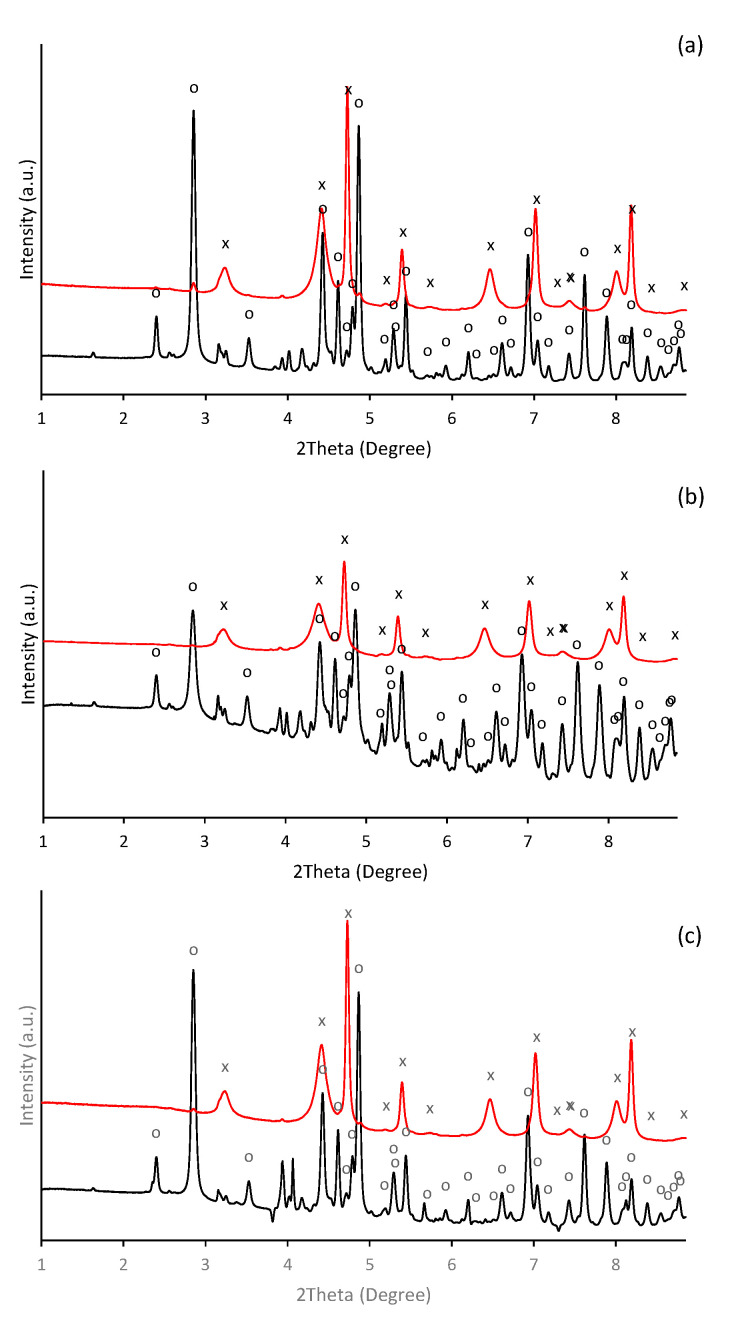
Diffraction patterns of the ferric precipitates maturated in alkaline (**a**) selenium-free, (**b**) selenite-treated, and (**c**) selenate-treated solutions collected on the 5th day of precipitate maturation (yellow line) and thermal transformation into hematite (blue line) (“o” and “x” symbols indicate characteristic XRD patterns for goethite (α-FeOOH) and hematite (α-Fe_2_O_3_), respectively).

However, the HEXRD analyses recognized, besides the presence of hematite (refined lattice parameters: a = 5.052(8) Å, c = 13.78(4) Å) as a major crystalline phase, also a residual amount of goethite in all treatments (Figure 8). Furthermore, the diffraction patterns exhibit relatively broad line characteristics for nanocrystalline materials. Since the dehydroxylation of goethite was found to be affected by the size of particles [62], the presence of its residues can be attributed to retention of water within the porous structure of only partially dehydroxylated goethite.

**Figure 9:**
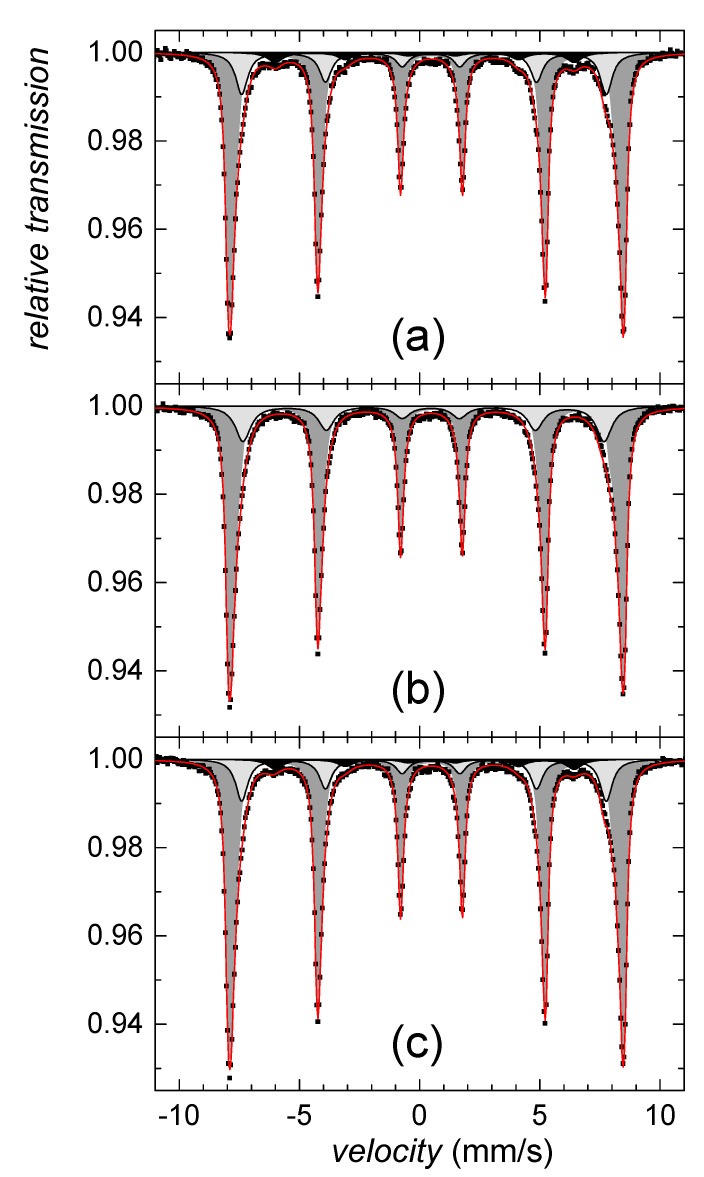
Mössbauer spectra of crystalized hematite from (**a**) selenium free, (**b**) selenite-treated and (**c**) selenate-treated solutions. Individual spectral components represent crystallized hematite phases; the well crystalized hematite (dark grey), less ordered hematite (light grey) and the highly disordered fraction (black).

The nanosized character of residual goethite crystals, and their dehydroxylation in various degrees, are most likely the reasons why the Mössbauer analysis has identified only its minor contribution (~5%) in room temperature spectra of selenium-free sample and after treatment with selenate. The corresponding spectral component (S6) is plotted in a black color in Figure 9a,c, respectively. No traces of goethite were found in the Mössbauer spectrum of thermally transformed selenite-treated samples. All spectral parameters derived from Mössbauer analyses are listed in Table 2. Major components of the Mössbauer spectra consist of two structurally different phases, comprising well crystalized hematite (S1–S4) and less ordered hematite (S5). They are shown in Figure 9 in a dark and light grey color, respectively. The Mössbauer data on distribution of the structurally different irons in thermally transformed precipitates revealed it was affected by the selenium presence.

The distribution of well crystalized hematite, less ordered hematite, and goethite fraction in thermally transformed precipitates was 76%, 18% and 6% for selenium-free precipitates, 81%, 19%, and 0% for selenite-treated precipitates, and 79%, 16% and 5% for selenate-treated precipitates, respectively. Due to statistical errors, there is only a marginal difference between the selenium-free and selenate-treated samples. However, selenite-treated sample did not comprise of highly disordered iron fraction. We hypothesize that this deviation is due to lesser goethite crystal size in comparison to other treatments (Figure 4b), which allowed the sample transformation into more ordered phases in shorter time.

### 2.4. Selenite and Selenate Interaction with Ferric Iron Precipitates

Co-precipitation of selenite and selenate with iron ferric phases has been established as a promising, easy-to-implement technology that carries various economic benefits, and is supposedly highly efficient [33]. However, there are some limitations. Our data indicate that the co-precipitation is successfully applicable only for highly diluted selenium-contaminated and slightly acidic aqueous media, where selenium concentration level is per million or billion, and the iron dose is of grams per liter [32].

In our experiment, the molar ratio of selenium and iron is approximately 1:1, and pH is over 12. The highly alkaline nature of suspension is unfavorable for selenium removal by freshly precipitated ferric oxides and hydroxides [63]. It seems that the higher density of positive-net charge at low pH is crucial for the initiation of adsorption, and stabilization of selenium on the ferric phases’ surfaces [36]. Even though no statistically significant high selenium removal has been observed in our experiment (data not shown), selenium is associated with the precipitates’ surfaces under these conditions. The observed selenium concentration shifts in course of time during precipitation and ferric phases’ maturation were very close within the estimated uncertainty of measurements (3%). It only highlights that the adsorptive interaction is negligible regarding concentration of remaining free selenium in suspension.

The SEM-EDS analysis of the ferric phases surely indicated the presence of selenium at the precipitates’ surfaces (Table 3). The results also indicated the presence of impurities in used chemicals (e.g., Mo and Si). The apparent surface coverage on the two- and five-day-old precipitates ranged from 0.9% to 3.6% with no statistically significant differences between the sampling periods. However, since it was reported by Zelmanov and Semiat [64] that even the nano-sized iron phases failed to adsorb selenite and selenate over the pH values of 10 and 8.3, respectively, we suspected that the observed Fe–Se association will not be stable. The adsorption kinetics in our previous work indicated that the selenium binding on the goethite surfaces is pH sensitive and highly labile in alkaline solutions [36]. Therefore, at pH 12, the negligible values of Fe–Se binding energies and reversible adsorption are expected. At this pH we hypothesize that mechanism of selenium bonding on surface is based on the weak electrostatic interactions. The main mechanism might involve the outer-sphere complexation.

**Figure 10:**
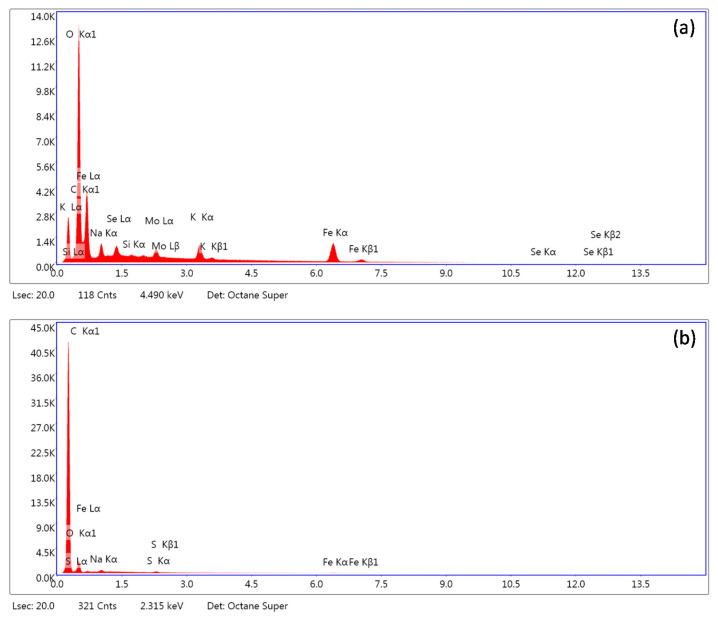
SEM-EDS analysis of selenium-bearing ferric phases (**a**) before and (**b**) after desorption using distilled water.

Figure 10 highlights that after applying sonication in distilled water on precipitates for selenium desorption, virtually no selenium was detected at the ferric iron mineral surfaces. It confirmed the hypothesized formation of highly labile Fe–Se associations during precipitation. The unstable selenium binding most likely resulted from a repulsive nature of the electrostatic interactions between the precipitates’ surfaces and selenium at pH 12. Since the ferric iron minerals’ point of zero charge usually lays below 8.5 [65,66], the presence of the dominant negative surface net-charge restricted the chemical binding of selenium oxyanions at the sorption sites (e.g., formation of inner-sphere complexes) [67]. Therefore, it is very likely that in our case the adsorption is governed by immobilization of selenium in the diffusion layer where it is unstable and easily removable after dilution of the bulk solution. Thus, the desorption of selenium from the collected precipitates using distilled water was highly efficient.

Although the free selenium concentration level in suspension did not change statistically significantly throughout the whole experiment, the content of free iron decreased extremely due to precipitation and maturation of ferric iron mineral phases. On the day after experiment initiation, the remaining free iron in solution (separated by centrifugation from precipitates) accounted for only up to 0.02% of the initial iron content. This was expected since the iron (co)precipitation in solutions containing stoichiometric or excessive amounts of strong alkali compounds (such as KOH) is one of the recommended methods for quantitative synthesis of iron-based materials [68].

**Figure 11:**
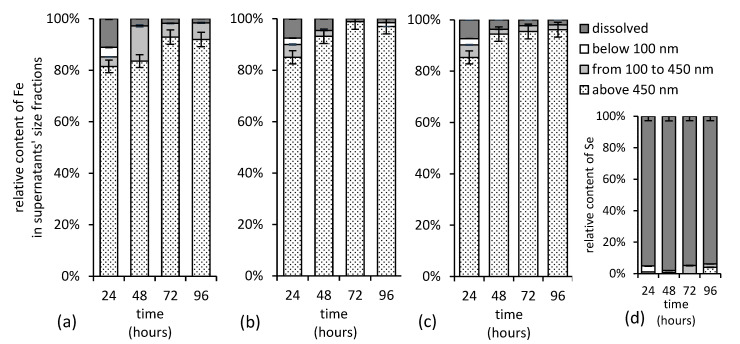
Concentration of iron in various supernatant’s size fractions separated from alkaline (**a**) selenium-free, (**b**) selenite-treated, and (**c**) selenate-treated suspensions and (**d**) concentration of selenite in solution during maturation of ferric iron precipitates. Both, selenium and iron, were determined in supernatants using F AAS.

Since there were no statistically significant differences in iron and selenium solution concentrations after the initial 24 h, the size fractionation has been performed. The experimental data presented in Figure 11 suggest that the high concentration of selenium do not favor the formation of its stable associations with (colloidal) iron in solution. Iron in solution clearly did not affect the selenium distribution, since over 95% of selenium was detected in the dissolved fraction (Figure 11d), while iron was abundant in colloidal fraction with dimension over 0.45 µm (Figure 11a–c) regardless of the treatment. Thus, we concluded that there was no consequential interaction between the colloidal iron and selenium, and, thus, the selenium oxyanions did not strongly associate with any particulate iron form at pH 12.

## 3. Materials and Methods

### 3.1. Chemicals and Reagents

The stock solutions of chemicals used in this study were prepared from Na_2_SeO_3_ (Lachema, Brno, Czech Republic), Na_2_SeO_4_ (Lachema, Brno, Czech Republic), KOH (Centralchem, Bratislava, Slovakia), Fe(NO_3_)_3_·9H_2_O (Alfa Aesar, Haverhill, MA, USA) and redistilled H_2_O. All chemicals were of analytical grade and were used as received without any further purification.

### 3.2. Solid Phase Preparation and Selenium Desorption

The method of Böhm referenced in Schwertmann and Cornell [42] was used for synthesis of goethite. An amount of 1.77 g Na_2_SeO_3_ or 1.88 g Na_2_SeO_4_ was dissolved in a volume of 18 mL of 5 mol L^−1^ KOH in a polyethylene flask. The solution was mixed with a 10 mL of 1 mol L^−1^ Fe(NO_3_)_3_ and immediately diluted to 200 mL with redistilled water. The selenium-free control was also prepared accordingly. The flasks were sealed, and the precipitate was left to age at pH 12 and at 70 °C in the laboratory oven (Memmert, Büchenbach, Germany).

The suspension with formed precipitates was mixed once a day during five-day aging, and the samples of precipitates from selenium-free, selenite, and selenate treatments were collected daily, centrifuged for 25 min at 5000 rpm (Eppendorf 5804R, Eppendorf, Hamburg, Germany), and then lyophilized. The samples of each treatment collected on the fifth day were also filtered, dried under 100 °C for an hour in the oven, and thermally transformed into hematite at 250 °C for 4 h. Afterwards, all samples were ground in agate mortar and stored in a sealed plastic tubes at room temperature for further analyses.

The desorption experiment was performed with the suspension of 100 mg of the selenate-treated precipitate, and 8 mL distilled H_2_O was sonicated for 10 min and then centrifuged at 4000 rpm, 2 times for 20 min. Supernatant was removed and solid phase was then again sonicated for 10 min and centrifuged at 4000 rpm, 2 times for 20 min. The suspension of the product and 2 mL distilled H_2_O was subsequently dried at 90 °C. Dried samples were then put in the desiccator. Samples after such treatment were then further analyzed.

### 3.3. Analytical Procedures

Ferric oxyhydroxides transformation and the effect of selenite and selenate on precipitates were analyzed by high energy X-ray diffraction (HEXRD). The experiment was performed at beamline P02.1 of PETRA III electron storage ring (DESY, Hamburg, Germany). The energy of the beam was set to *E* = 59.73091 keV (*λ* = 0.20757 Å). The samples were put into Kapton tubes (Broomall, PA, USA) with a diameter of 1 mm and were illuminated by an incident beam having a cross section of 0.5 mm× 0.5 mm. All the diffraction patterns were collected at room temperature using a two-dimensional detector Perkin Elmer 1621 (2048 × 2048 pixels, pixel size 200 μm × 200 μm). The sample-to-detector distance (SDD) was set to 1285 mm. A CeO_2_ standard was used to calibrate SDD and relative tilt of the detector to the incident beam path. Two-dimensional diffraction patterns were integrated using FIT2D software (Grenoble, France).

Mössbauer spectroscopy, which enabled unique identification of different iron sites, their valence states, and the type of magnetic order during aging and transformation of ferric precipitates, was also applied. Mössbauer spectrometer was equipped with a ^57^Co/Rh radioactive source, and it was operated in transmission geometry at room temperature. The velocity scale was calibrated with a metallic iron foil (Goodfellow, Huntingdon, UK, thickness 12.5 µm). Isomer shift values are quoted with respect to that of a Mössbauer spectrum of α-Fe recorded at room temperature.

The Fourier transform infrared spectroscopy-attenuated total reflectance (ATR-FTIR) measurements of samples were performed with Nicolet 400 FTIR spectrometer (Thermo Scientific, Waltham, MA, USA) using a single bounce ATR accessory equipped with a germanium crystal. For each measurement, spectral resolution of 2 cm^−1^, a scan range was 4000–400 cm^−1^ and 64 scans per analysis was performed.

To improve the understanding of selenium sorption mechanisms, a selenate-treated sample from the second day of maturation was washed with distilled water, while the detailed procedure was conducted as described above. After the removal of weakly bonded compounds, the samples were analyzed by scanning electron microscopy (SEM) and energy-dispersive X-ray spectroscopy (EDS) in order to obtain information about iron and selenium content. All the micrographs were acquired by scanning electron microscope Tescan FERA 3 (Brno, Czech Republic) equipped with Octane Super 60 mm^2^ EDS system.

### 3.4. Iron and Selenium Size Fractionation

Fractionation and separation of particles in solution were performed by 4 steps: centrifuge at 5000 rpm for 25 min, filtration through the filter with membrane filters with pore size of 0.45 µm and 0.1 µm and ultracentrifugation. Determination of selenium and iron in the aqueous phases collected during the experiment was performed using a flame atomic absorption spectrometer (F AAS) model Perkin Elmer, Waltham, MA, USA, 1100 in air-acetylene flame. As a radiation sources, for selenium and iron were EDL 2 (electrodeless discharge lamp), HCL (hollow-cathode lamp), respectively. A solution of selenium and iron standards were prepared in 0.1 mol L^−1^ KNO_3_. The calibration range for selenium determination using F AAS was 0–50 mg L^−1^ and for iron 0–10 mg L^−1^. Samples containing higher concentration were diluted. Calibration standards were prepared from a stock solution of 1000 mg L^−1^ (Merck CertiPur, Darmstadt, Germany) for both elements. The limit of detection (LOD) was 0.1 mg L^−1^, and the limit of quantification (LOQ) was 0.3 mg L^−1^ for selenium and 0.01 mg L^−1^ and 0.03 mg L^−1^ for iron.

## 4. Conclusions

This study highlights that the precipitation of ferric oxyhydroxides is a relatively fast process, where the initial kinetics depend on the chemical speciation of selenium. Selenite and selenate were most probably bound by weak physical interactions with goethite surfaces and, thus, they do not manifest themselves in the mineralogy of the precipitates since goethite was observed as the only major phase. Surprisingly, no evidence of ferrihydrite was indicated. Although there were no changes in mineralogy induced by the presence of selenium, it slightly altered morphology and crystallinity of precipitated goethite grains. The results suggested indistinct inhibition of crystal growth in the selenite-treated precipitate that caused the slight effect on goethite’s needle-like particles morphology in comparison to both selenium-free and selenate treatments. It was proven that relatively high concentrations of selenium and alkaline pH led to stabilization of the nano-sized particles of goethite during its crystallization, which effected its removal capacity. Mössbauer analysis also indicated alteration in the crystallinity of ferric precipitates, and it showed that the presence of selenite and selenate interrupted maturation at the initial phase. However, crystal integrity and homogeneity increased with time in each treatment. We also observed significant changes in crystallinity of hematite in the presence of selenite. However, morphology and ATR-FTIR spectra of hematite appeared to be similar, with only marginal changes in all treatments. These results provide unique insight into selenium effects on the structural changes of nanosized ferric phases during their precipitation, maturation, and thermal transformation into hematite at alkaline conditions. The stability and indistinct changes of iron oxyhydroxides show the suitability of the iron co-precipitation method for contaminant removal from selenium-contaminated waters at alkaline pHs and increased temperatures.

## Figures and Tables

**Table 1:** Mössbauer parameters obtained from room temperature spectra comprising distributed component (dist) and two sextets (S1, S2): relative spectral area, *A_rel_*, hyperfine magnetic field, *B_hf_*, isomer shift, *δ*, and quadrupole shift, *ε*_Δ_. The estimated experimental errors are of ±1.5%, ±0.3 T, ±0.02 mm/s, and ±0.04 mm/s, correspondingly.

Sample		2nd Day of Precipitation			5th Day of Precipitation		
Spectral Component		Dist	S1	S2	Dist	S1	S2
	Parameter						
Selenium-free	*A_rel_* (%)	51.7	29.4	18.9	47.6	30.9	21.5
	*B_hf_* (T)	28.6	38.4	37.4	31.2	38.1	37.2
	*δ* (mm/s)	0.35	0.37	0.24	0.35	0.37	0.24
	*ε*_Δ_ (mm/s)	−0.28	−0.28	−0.28	−0.28	−0.27	−0.27
Selenite-treated	*A_rel_* (%)	60.0	22.2	17.8	51.5	27.6	20.9
	*B_hf_* (T)	29.4	38.0	37.0	31.1	38.0	37.1
	*δ* (mm/s)	0.35	0.37	0.24	0.35	0.37	0.24
	*ε*_Δ_ (mm/s)	−0.29	−0.27	−0.27	−0.28	−0.27	−0.27
Selenate-treated	*A_rel_* (%)	56.2	27.2	16.6	47.5	31.9	20.6
	*B_hf_* (T)	28.9	38.1	37.1	30.7	38.1	37.2
	*δ* (mm/s)	0.35	0.37	0.24	0.35	0.37	0.24
	*ε*_Δ_ (mm/s)	−0.29	−0.28	−0.28	−0.28	−0.27	−0.27

**Table 2:** Mössbauer parameters obtained from room temperature spectra of thermally transformed samples comprising the S1–S6 sextets: relative spectral area, *A_rel_*, hyperfine magnetic field, *B_hf_*, isomer shift, *δ*, and quadrupole shift, *ε*_Δ_. The estimated experimental errors are of ±1.5%, ±0.3 T, ±0.02 mm/s, and ±0.04 mm/s, correspondingly.

Sample		Thermal			Transformation		
Spectral Component		S1	S2	S3	S4	S5	S6
	Parameter						
Selenium-free	*A_rel_* (%)	31.3	27.3	12.1	5.4	18.4	5.5
	*B_hf_* (T)	51.3	50.5	49.6	48.5	47.1	38.6
	*δ* (mm/s)	0.38	0.39	0.37	0.37	0.32	0.36
	*ε*_Δ_ (mm/s)	−0.21	−0.21	−0.23	−0.27	−0.29	−0.30
Selenite-treated	*A_rel_* (%)	36.3	27.0	11.6	5.6	19.5	-
	*B_hf_* (T)	51.2	50.3	49.2	47.9	46.6	-
	*δ* (mm/s)	0.38	0.38	0.38	0.35	0.31	-
	*ε*_Δ_ (mm/s)	−0.21	−0.21	−0.25	−0.25	−0.30	-
Selenate-treated	*A_rel_* (%)	31.5	28.8	12.7	6.0	16.4	4.6
	*B_hf_* (T)	51.3	50.5	49.5	48.4	46.8	38.9
	*δ* (mm/s)	0.38	0.39	0.37	0.36	0.32	0.34
	*ε*_Δ_ (mm/s)	−0.21	−0.21	−0.24	−0.27	−0.30	−0.35

**Table 3:** Selenium and iron content at the surfaces of the ferric precipitates (%) indicated by the SEM-EDS analysis of the representative areas of the samples collected on the 2nd and 5th day of precipitate maturation, and after its thermal transformation into hematite (data indicate an average value of the three representative areas with standard deviation). The asterisks indicate a statistically significant difference between the treatments at the (*) 0.05 and (**) 0.01 levels.

Sample	Element (Weight %)	2nd Day of Precipitation	5th Day of Precipitation	Thermal Transformation
Selenium-free	Fe	26.8 ± 3.2	26.2 ± 2.3	33.2 ± 1.3 *
Selenite-treated	Fe	25.7 ± 1.9	25.2 ± 1.9	25.5 ± 2.5
	Se	2.3 ± 0.9	1.5 ± 0.1	0.93 ± 0.08 **
Selenate-treated	Fe	25.4 ± 2.5	26.1 ± 0.37	36.01 ± 2.04 *
	Se	2.5 ± 1.0	1.1 ± 0.20	0.95 ± 0.1
